# Untargeted Metabolomics Reveals Distinct Anthocyanin Profiles in Napier Grass (*Pennisetum purpureum* Schumach.) Cultivars

**DOI:** 10.3390/foods14152582

**Published:** 2025-07-23

**Authors:** Zhi-Yue Wang, Pei-Yin Lin, Chwan-Yang Hong, Kevin Chi-Chung Chou, Ting-Jang Lu

**Affiliations:** 1 Institute of Food Science and Technology, National Taiwan University, Taipei 10617, Taiwan; d10641007@ntu.edu.tw (Z.-Y.W.); kevinchou@ntu.edu.tw (K.C.-C.C.); 2 Joint Center for Instruments and Researches, College of Bioresources and Agriculture, National Taiwan University, Taipei 10617, Taiwan; zpylin@ntu.edu.tw; 3 Department of Agricultural Chemistry, National Taiwan University, Taipei 10617, Taiwan; cyhong@ntu.edu.tw; 4 Agricultural Experimental Farm, College of Bioresources and Agriculture, National Taiwan University, Taipei 10617, Taiwan

**Keywords:** Poaceae plants, napier grass, plant secondary metabolites, anthocyanins, UHPLC-HRMS

## Abstract

Plant secondary metabolites regulate plant growth and serve as valuable pharmaceutical resources. Napier grass (*Pennisetum purpureum* Schumach.), a Poaceae species, shows potential as a functional food. In this study, we employed high-resolution mass spectrometry combined with a data-independent acquisition (DIA) strategy for the untargeted detection of anthocyanins, a group of secondary metabolites, in napier grass. Clear MS^2^ fragmentation patterns were observed for anthocyanins, characterized by diagnostic aglycone signals and sequential losses of hexosyl (C_6_H_10_O_5_), deoxyhexosyl (C_6_H_10_O_4_), pentosyl (C_5_H_8_O_4_), and *p*-coumaroyl groups (C_9_H_8_O_3_). Based on matching with authentic standards and an in-house database, ten anthocyanins were identified, seven of which were newly reported in napier grass. In a single-laboratory validation analysis, both absolute and semi-quantitative results reliably reflected the specific distribution of metabolites across different cultivars and plant organs. The purple cultivar (TS5) exhibited the highest anthocyanin content, with the cyanidin 3-*O*-glucoside content reaching 5.0 ± 0.5 mg/g, whereas the green cultivar (TS2), despite its less pigmented appearance, contained substantial amounts of malvidin 3-*O*-arabinoside (0.7 ± <0.1 mg/g). Flavonoid profiling revealed that monoglycosylated anthocyanins were the dominant forms in floral tissues. These findings shed light on napier grass metabolism and support future Poaceae breeding and functional food development.

## 1. Introduction

*Pennisetum purpureum* Schumach., commonly known as napier grass, belongs to the Poaceae family and is widely recognized as an important forage crop, with reported antibacterial [1,2], antidiabetic [3], antioxidant [1,2], and antifungal [2,3] properties. Notably, the U.S. FDA approved clinical trials of a *P. purpureum*-based botanical drug candidate for cancer treatment (coded as ZR01—see Table S1), with associated patents for its anticancer bioactive compounds and pharmaceutical applications. Recently, Ho et al. highlighted the potential of anthocyanin-rich purple napier grass extract to effectively alleviate high-fat diet-induced obesity and metabolic disorders via the modulation of the PI3K/Akt and AMPK pathways, further supporting its health-promoting value [4]. As a non-traditional edible material, *P. purpureum* has gained popularity as an eco-friendly functional ingredient. Despite these promising developments, our understanding of its chemical composition—particularly the bioactive components of *P. purpureum* and its fermented products—remains limited. This lack of comprehensive data poses a major obstacle to its high-value development as functional products.

Phytochemical screening has identified seven major classes of secondary metabolites in *P. purpureum*, including alkaloids, cyanogenic glycosides, flavonoids, oxalates, phytates, saponins, and tannins [5]. These findings suggest that *P. purpureum* and its related species possess a broad range of nutritional and bioactive properties. However, the specific functional compounds responsible for these effects have yet to be fully elucidated. Additionally, the stems and leaves of the purple cultivar exhibit a reddish coloration due to the high accumulation of anthocyanins, which may serve as a visual indicator of its strong antioxidant potential [6].

Anthocyanins are non-toxic phenolic compounds responsible for the red, purple, and blue pigmentation in many flowers, fruits, and vegetables [7]. Structurally, anthocyanins are an anthocyanidin aglycone linked to one or more sugar moieties. Because of variability in glycosylation patterns and substitution sites on the aglycone, many structural isomers are formed [8]. This structural diversity not only underlies the wide range of biological and physiological activities attributed to anthocyanins but also poses a significant challenge to their identification using conventional analytical methods, which often suffer from limited efficiency.

Onjai-Uea et al., reported the presence of anthocyanins in purple cultivars; however, due to limitations in regard to analytical tools, the characterization of individual anthocyanin components remained insufficient [9]. Zhou et al., annotated potential anthocyanins in napier grass based on mass-spectrometry data matched with online databases [10]. Nevertheless, their identification was based primarily on matches with online spectral databases, which often provide limited information that may vary depending on the instrument platform [11]. Without confirmation according to chromatographic retention times and MS/MS fragmentation patterns, metabolite annotations remain tentative.

To overcome these limitations, in this study, we employed an advanced approach coupling ultra-high-performance liquid chromatography (UHPLC) with high-resolution mass spectrometry (HRMS). The UHPLC system enabled the efficient separation of isomeric compounds, while a Q-Exactive Orbitrap mass spectrometer enabled high-resolution and high-accuracy mass detection and the performance of simultaneous qualitative and quantitative analyses in a single run, improving the accuracy of structural elucidation and quantification in complex plant extract mixtures, streamlining the compound isolation process, and facilitating natural-product research.

We also employed a suspect-screening strategy by constructing an in-house structural database of 158 anthocyanins derived from flowers, vegetables, and fruits. This approach addresses common challenges in plant metabolomics, such as the limited coverage of public spectral databases and the slow pace of metabolite isolation and purification. This method provides critical technical support for elucidating the bioactive compounds in napier grass by enabling high-throughput identification of its complex anthocyanin profiles, laying a foundation for the development of visually appealing, health-promoting food products. This work builds upon this analytical framework to characterize the diversity and distribution of anthocyanin metabolites in napier grass, providing a basis for future studies on their biological relevance.

## 2. Materials and Methods

### 2.1. Materials

The cultivars of napier grass used in this study were Taishiu No. 2 (TS2), No. 5 (TS5), and No. 6 (TS6). TS2 and TS6 samples were obtained from local farmers in Taiwan (22.5495° N, 120.62° E). Samples of TS5, including leaves, stems, and inflorescences, were provided by the Agricultural Experimental Farm of the College of Bioresources and Agriculture, National Taiwan University (25.0154° N, 121.5401° E). Detailed information on these cultivars is provided in Supplementary Materials Table S2.

Approximately twenty individual plants were sampled per cultivar for each type of tissue (inflorescence, leaf, and stem). Equal amounts of each tissue were pooled to generate a single composite sample representing the population-level metabolite profiles for each cultivar and tissue. From this homogenized pool, three technical replicates were randomly subsampled and analyzed. All plant materials were lyophilized, ground, and stored at −20 °C before analysis.

### 2.2. Chemicals and Reagents

Cyanidin 3-*O*-glucoside (CAS number: 7084-24-4; purity ≥ 95.0%) and peonidin 3-*O*-glucoside (CAS number: 6906-39-4; purity ≥ 95.0%) standards were purchased from Merck (Darmstadt, Germany), while malvidin (CAS number: 643-84-5; purity ≥ 98%) and malvidin 3-*O*-arabinoside (CAS number: 679429-95-9; purity ≥ 98%) standards were purchased from Chemface (Wuhan, PRC). LC-MS-grade acetonitrile was purchased from J.T. Baker (Phillipsburg, NJ, USA); ethanol (purity ≥ 95%) was purchased from Jing Ming Chemical Co., Ltd., in Miaoli, Taiwan; and formic acid (purity of 99%, ACS grade) was purchased from Honeywell Fluka (Muskegon, MI, USA). Deionized water (Purelab Ultra Genetic, TOC < 1 ppb) with a resistivity of ≥18.2 MΩ·cm at 25 °C was obtained from ELGA (Lane End, High Wycombe, UK).

### 2.3. Sample Preparation

First, 0.5 g of the lyophilized sample powder was added to a 50 mL centrifuge tube, followed by 20 mL of 1% HCl in 80% methanol. The mixture was vortexed for 30 s and then sonicated in an ultrasonic bath for 1 h before being centrifuged at 2350× *g* for 5 min, both conducted at 25 °C. The entire supernatant was collected and transferred to another 50 mL centrifuge tube. Subsequently, caffeine was added at a final concentration of 0.05 μg/g as an internal standard. The solution was then filtered through a 0.22 μm polyvinylidene difluoride (PVDF) membrane filter and transferred into a 2 mL amber vial for LC-MS/MS analysis. Surrogate samples were prepared by mixing lyophilized powders of anthocyanin-rich fruits and flowers and then carrying out extraction using the same procedure employed for the napier grass samples.

### 2.4. Instruments and Conditions

The anthocyanin compositions of the selected samples were analyzed using UHPLC coupled with diode array detection (DAD) and a high-resolution mass spectrometer equipped with an electrospray ionization (ESI) source. The analytical system included an UltiMate™ 3000 rapid-separation autosampler and an UltiMate™ 3000 rapid-separation photodiode array (PDA) detector, supplied by Thermo Fisher Scientific (Waltham, MA, USA). A mass-spectrometric analysis was performed using a Q-Exactive mass spectrometer, with data processing carried out using Xcalibur 4.0 software from Thermo Fisher Scientific.

A Kinetex PFP column (2.1 mm × 150 mm, 1.7 µm) produced by Phenomenex (Torrance, CA, USA) was used for chromatographic separation, with the column temperature maintained at 30 °C. The mobile phases consisted of ultrapure water (A) and acetonitrile (B), each containing 1% (*v*/*v*) formic acid. The flow rate was maintained at 0.40 mL/min. Gradient elution was programmed as follows: 0–20 min, 3% B; 20–25 min, 17% B; 25–28 min, 70% B; 28–32min, 95% B; 32–33min, 95% B; and 33–40 min, 3% B.

Detection was performed using a diode array detector (DAD) with a 520 nm wavelength and a scanning range of 190–800 nm. The autosampler temperature was maintained at 5 °C, and the injection volume was 5 µL.

The mass-spectrometric analysis was conducted using an electrospray ionization (ESI) source in positive-ion mode. The instrument parameters were set as follows: source voltage, 3.5 kV; capillary temperature, 263 °C; S-lens RF level, 55; auxiliary gas heater temperature, 425 °C; sheath gas flow rate, 50 units; and auxiliary gas flow rate, 13 units.

In full-scan mode, the parameters were set to a full MS resolution of 700,000, a scanning range from 150 to 2250, and an automatic gain control (AGC) target of 1 × 10^6^. The data-dependent MS^2^ (ddMS^2^) settings included a resolution of 35,000; step-normalized collision energy (NCE) values of 15, 35, and 55; an AGC target of 2 × 10^5^; a maximum injection time (IT) of 100 ms; and an isolation window of 2.0 *m*/*z*. The diisooctyl phthalate peaks at 391.28429 [M + H]^+^ were used as a lock mass in positive-ion mode.

### 2.5. Identification Strategy

**Figure 1:**
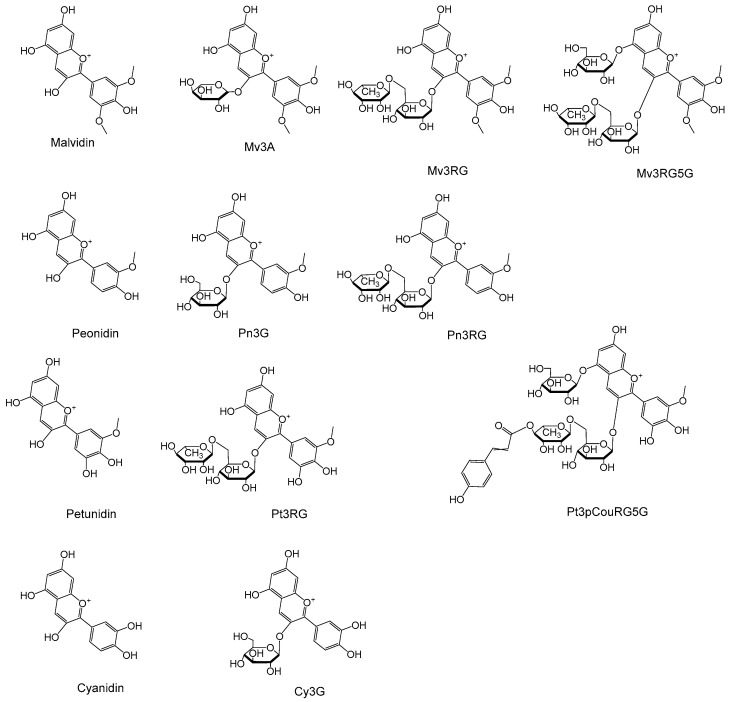
Anthocyanins and their aglycons in napier grass.

Untargeted metabolite profiling of napier grass was conducted using a data-independent acquisition (DIA) strategy, specifically, Full MS/dd-MS^2^ (Top10) acquisition mode, which generated many molecular features. These features were screened against an in-house database containing 158 anthocyanins (Table S3), and tentative structures were assigned accordingly. Among them, cyanidin, malvidin, cyanidin 3-*O*-glucoside (Cy3G), malvidin 3-*O*-arabinoside (Mv3A), and peonidin 3-*O*-glucoside (Pn3G) were confirmed through a comparison with authentic standards and thus classified as Level 1 identifications. The remaining compounds were annotated as Level 2, as they were based on mass accuracy, characteristic fragment ions, spectral matching with database entries, and comparison with surrogate samples. Identification confidence levels were assigned based on the criteria proposed by Schymanski et al., for high-resolution-mass-spectrometry-based metabolite identification [12]. Metabolite identification was conducted using the identification point system described in Commission Implementing Regulation (EU) 2021/808 (http://data.europa.eu/eli/reg_impl/2021/808/oj, accessed on 15 July 2025), which provides standardized criteria for confirming chemical substances in complex matrices. The structures of the anthocyanins and their corresponding aglycones identified in napier grass are shown in Figure 1.

### 2.6. Quantification Criteria

For quantification, the molecular features of the putative anthocyanins identified through untargeted analysis were further evaluated. Absolute quantification was performed for selected target compounds using authentic standards of Mv3A, Cy3G, and Pn3G. Other anthocyanins were quantified semi-quantitatively based on a relative response. All quantification results underwent single-laboratory validation, covering key parameters such as accuracy, sensitivity, linearity, reproducibility, precision, stability, and recovery. Therefore, the anthocyanin concentrations obtained are considered reliable and suitable for both absolute and relative comparisons across different samples.

Each calibration curve was established based on the least-squares linear regression model, using the integrated peak area of individual authentic standards. The linearity of all the calibration curves was evaluated using their coefficient of determination (R^2^) values. The lowest point of each calibration curve was defined as the reporting limit, with the average signal-to-noise (S/N) ratio exceeding 10.

The calibration curves and related characteristics of the three analytes are summarized in Table S4. All the standards exhibited correlation coefficients greater than 0.99, indicating a strong linear relationship across the calibration range.

The accuracy of the method was evaluated through recovery tests. Mv3A was spiked into the solvent at concentrations of 4.0 μg/g, 400.0 μg/g, and 1600.0 μg/g, while Cy3G and Pn3G were added at 4.0 μg/g, 40.0 μg/g, and 400.0 μg/g. The recovery rates ranged from 85.5 ± 7.4% to 113.7 ± 2.7%, indicating good accuracy (Table S5).

In this study, the stability and accuracy of the analytical platform were validated by measuring the concentrations and retention times of three anthocyanins in quality control (QC) samples. The QC samples were analyzed alongside the napier grass samples, resulting in 15 measurements (corresponding to three replicates per day over five consecutive days). These data were used to evaluate intra-day and inter-day precision. Intra-day precision was assessed by analyzing three replicate QC samples on the same day, while inter-day precision was evaluated over five consecutive days. Control charts were constructed based on the mean value ($\bar{X}$), which is shown as a red solid line in the figure, and the standard deviation (SD). Warning limits ($\bar{X\;}\pm 2SD$, dashed lines) and control limits ($\bar{X\;}\pm 3SD$, solid black lines) were also plotted (Figure S1).

For all 15 measurements, the concentrations of the three anthocyanins remained within the control limits, and the variation in retention time was less than 0.2 min. The intra-day precision (CV%) ranged from 0.002% to 0.138%, while the inter-day precision ranged from 2.397% to 2.778% (Table S6).

### 2.7. Differential Metabolites Selected

The acquisition and integration of total ion chromatograms were achieved using Xcalibur 4.0 software (Thermo Fisher Scientific). Extracted ion chromatograms (XICs) corresponding to the target metabolites were also obtained and integrated. All data points used for multivariate and clustering analyses represent the mean values of three technical replicates subsampled from pooled tissue samples, as described in Section 2.1. The peak area ratios (Area_anal_/Area_IS_) were normalized using a logarithm transformation prior to multivariate analysis. The normalized data were subsequently imported into the online OmicShare tools platform (http://www.omicshare.com/tools, accessed on 30 May 2025) for an orthogonal partial-least-squares discriminant analysis (OPLS-DA), with the threshold for variable importance in projection (VIP) set to 1; *p*-values less than 0.05 were considered statistically significant [13]. Heat maps with hierarchical clustering were generated using OriginPro 2021 (version 9.8.0.200, OriginLab Corporation, Northampton, MA, USA) with the Clustergram App. A logarithm transformation was applied to the data prior to clustering. Clustering was conducted on columns only, using the Euclidean distance and average linkage.

## 3. Results and Discussion

### 3.1. Qualitative Analysis of Anthocyanidins and Anthocyanins

**Figure 2:**
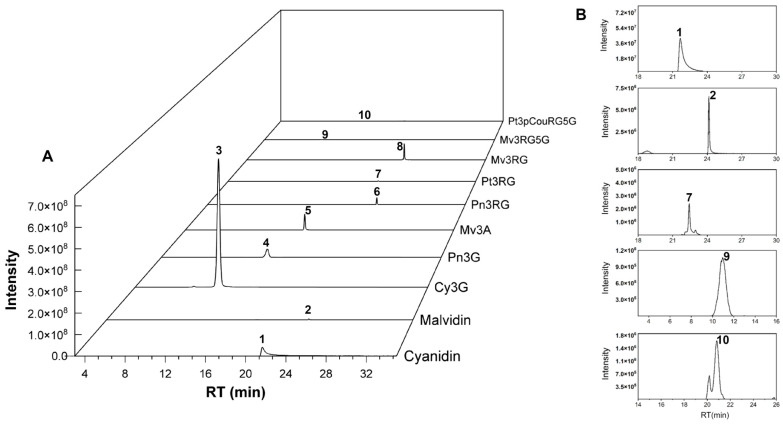
Extracted ion chromatograms (XICs) of (**A**) anthocyanins and (**B**) a magnified view of the selected region for the QC sample mixture. Peak 1: cyanidin; peak 2: malvidin; Peak 3: Cy3G, peak 4: Pn3G; peak 5: Mv3A, peak 6: Pn3RG; Peak 7: Pt3RG; peak 8: Mv3RG; Peak 9: Mv3RG5G; peak 10: Pt3pCouRG5G.

Ten anthocyanins were identified in the napier grass samples, consisting of anthocyanidins and their glycosylated derivatives, including mono-, di-, and triglycosylated forms, as well as *p*-coumaroylated anthocyanins (Figure 1). The extracted ion chromatograms of these compounds, generated via UHPLC-Orbitrap MS in the QC samples under optimized conditions, are shown in Figure 2.

The presence of four major anthocyanidin aglycones in the samples was confirmed by their characteristic signals at *m*/*z* 287.0550 (cyanidin), 301.0707 (peonidin), 317.0656 (petunidin), and 331.0807 (malvidin), serving as reliable diagnostic ions for identification. In nature, anthocyanins are commonly glycosylated, and the detection of neutral losses corresponding to sugar moieties—including hexose (162.053 Da), deoxyhexose (146.058 Da), and pentose (132.042 Da)—indicates the presence of mono- to highly glycosylated forms, sometimes exceeding five sugar units. In addition, acylation by hydroxycinnamic acid derivatives—such as *p-*coumaroyl groups (C_9_H_8_O_3_)—was observed, characterized by a neutral loss of 164.047 Da, which provided additional structural information. A list of the compounds identified in the napier grass samples is shown in Table 1.

Isomeric compounds such as Pn3G and Mv3A can be distinguished based on the identification rules described above, including by their aglycone types and glycosylation patterns, as well as their distinct retention times during chromatographic separation (Figure 2).

**Figure 3:**
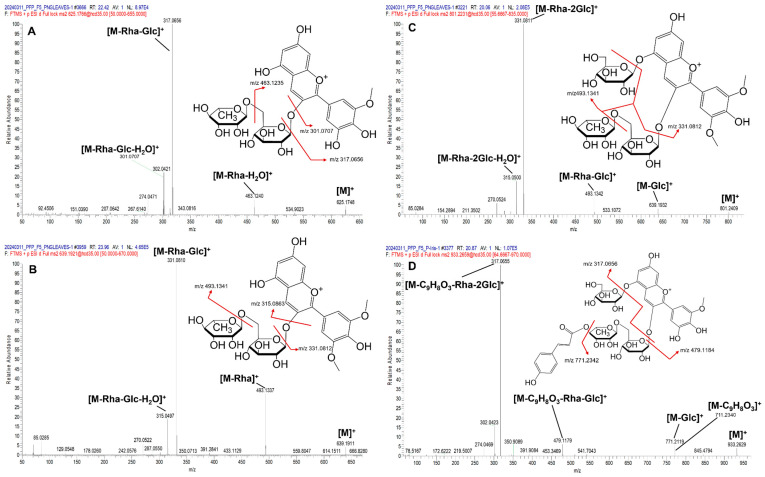
MS/MS spectra of (**A**) Pt3RG, (**B**) Mv3RG, (**C**) Mv3RG5G, and (**D**) Pt3pCouRG5G.

In the case of petunidin 3-*O*-rutinoside (Pt3RG) (C_28_H_33_O_16_^+^) (Figure 3A), the precursor ion appeared at *m*/*z* 625.1763 ([M]^+^). The fragment ions appeared at *m*/*z* 463.1240, corresponding to the loss of a rhamnose residue and water ([M − Rha − H_2_O]^+^); at *m*/*z* 317.0656, representing the loss of both rhamnose and glucose residues ([M − Rha − Glc]^+^); and at *m*/*z* 301.0707, indicating an additional loss of water ([M − Rha − Glc − H_2_O]^+^). Similarly, malvidin 3-*O*-rutinoside (Mv3RG) exhibited a characteristic fragment ion at *m*/*z* 331.0810, corresponding to the loss of rhamnose and glucose residues ([M − Rha − Glc]^+^) (Figure 3B).

In the case of trisaccharide anthocyanins such as malvidin 3-*O*-rutinoside-5-*O*-glucoside (Mv3RG5G) (Figure 3C), sequential sugar losses were observed in the MS^2^ spectra. The ion at *m*/*z* 639.1932 corresponded to the loss of one glucose residue ([M − Glc]^+^), while the ion at *m*/*z* 493.1342 represented the loss of both a rhamnose and glucose residue ([M − Rha − Glc]^+^). The ion at *m*/*z* 331.0811 indicated the loss of one rhamnose and two glucose residues ([M − Rha − 2Glc]^+^), and the ion at *m*/*z* 315.0500 corresponded to the additional loss of a water molecule ([M − Rha − Glc − H_2_O]^+^).

*p*-coumaroylated anthocyanins were also detected, including petunidin 3-*O*-(*p-*coumaroyl)rutinoside-5-*O*-glucoside (Pt3pCouRG5G). In the MS^2^ spectrum, the ion at *m*/*z* 771.2119 corresponded to the loss of a glucose residue ([M − Glc]^+^), while the fragment at *m*/*z* 479.1179 indicated the loss of a *p*-coumaroyl group (C_9_H_8_O_3_), a rhamnose residue, and a glucose residue ([M − C_9_H_8_O_3_ − Rha − Glc]^+^). The ion at *m*/*z* 317.0655 indicated the further loss of an additional glucose residue, corresponding to [M − C_9_H_8_O_3_ − Rha − 2Glc]^+^ (Figure 3D).

**Figure 4:**
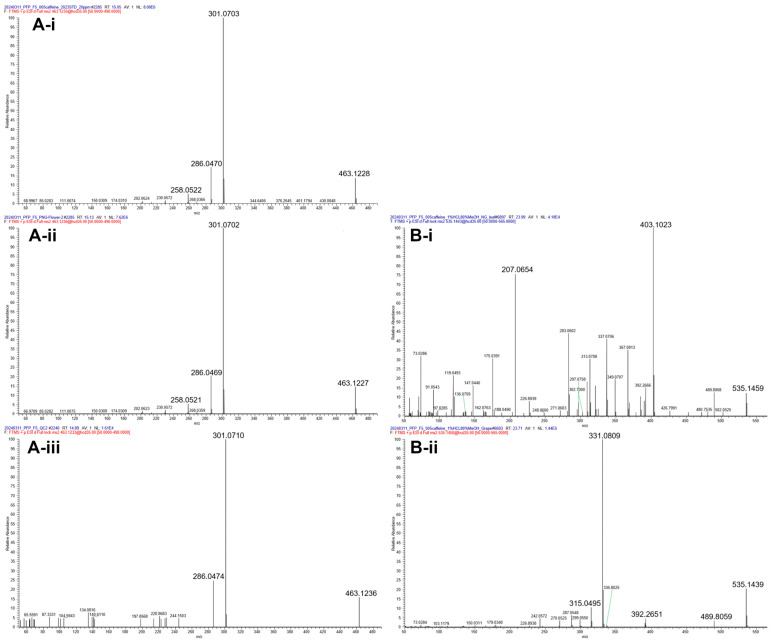
(**A**) Matched MS^2^ spectra of Pn3G in the standard (**A-i**), napier grass (**A-ii**), and surrogate (**A-iii**) samples, showing consistent fragmentation patterns and similar retention times; (**B**) no matched MS^2^ spectra were obtained for the ion at *m*/*z* 535.1440 detected in napier grass (**B-i**). The MS^2^ spectrum obtained from the surrogate samples (**B-ii**) matched that of malvidin 3-(6′′-acetylglucoside). The *y*-axis represents relative abundance, and the absolute intensity is indicated by the NL value shown in each spectrum.

In this study, we established a highly reliable compound identification approach using an in-house database supported by mass spectra from surrogate samples. In Figure 4A, panels A-i, A-ii, and A-iii represent the MS/MS spectra of peonidin Pn3G from the standard, napier grass, and surrogate samples, respectively. The fragment ions exhibited were consistent across the spectra, and their similar retention times (RT = 15.05, 15.13, and 14.89 min) further confirm their alignment. Given the lack of retention time (RT) information and potential interference from isomeric compounds, the possibility that some errors occurred in the identification of the secondary metabolites cannot be excluded. Previous attempts at elucidating the anthocyanin composition in napier grass using online database matching are inaccurate when compared to our validated method of detection. For example, Zhou et al., predicted the presence of compounds such as malvidin 3-(6′′-acetylglucoside) and pelargonidin 3-(6′′-acetylglucoside) in napier grass [10]; however, these compounds were not detected in our samples. Figure 4B shows that the ion at *m*/*z* 535.1440 was detected at retention times of 23.99 min and 23.71 min but exhibited inconsistent MS/MS fragmentation patterns. The fragmentation pattern at 23.71 min (Figure 4B-ii) detected in the surrogate samples matched that of malvidin 3-(6″-acetylglucoside), which was characterized by a fragment ion at *m*/*z* 331.0809 corresponding to [M − GlcAc]^+^ [14].

**Table 1:** List of compounds identified in napier grass.

Peak NO	Tentative Identification	RT (min)	Molecular Formula	[M]^+^ Theoretical *m*/*z*	[M]^+^ Observed *m*/*z* (Mass Error)	Characteristic Fragment Ion	UV/Vis Absorption Bands (nm)	Level	Ref.
1	Cyanidin	21.67	C_15_H_11_O_6_^+^	287.0550	287.0545 (1.7)	269.0444 [M-H_2_O]^2•+^ 241.0495 [M-CO-H_2_O]^2•+^ 213.0546 [M-2CO-H_2_O]^2•+^ 137.0233 [M-C_6_H_4_-2CO-H_2_O]^2•+^	520	1	[15,16]
2	Malvidin	23.90	C_17_H_15_O_7_^+^	331.0807	331.0813 (1.8)	315.0496 [M-CH_3_-H]^2•+^ 270.0523 [M-CH_3_OH-COH]^•+^ 242.0571 [M-CH_3_OH-COH-CO]^•+^	520	1	[15,17]
3	Cy3G	12.15	C_21_H_21_O_11_^+^	449.1078	449.1072 (1.3)	287.0545 [M-Glc]^+^	235,520,280	1	[15]
4	Pn3G	16.32	C_22_H_23_O_11_^+^	463.1235	463.1229 (1.3)	301.0703 [M-Glc]^+^	234,520	1	[15]
5	Mv3A	17.22	C_22_H_23_O_11_^+^	463.1235	463.1236 (0.2)	331.0809 [M-Ara]^+^	234,520,350	1	[15]
6	Pn3RG	24.04	C_28_H_33_O_15_^+^	609.1814	609.1814 (0.0)	463.1231 [M-Rha]^+^ 301.0702 [M-Rha-Glc]^+^	235,520	2	[17,18]
7	Pt3RG	22.49	C_28_H_33_O_16_^+^	625.1763	625.1764 (0.1)	463.1240 [M-Rha-H_2_O]^+^ 317.0656 [M-Rha-Glc]^+^ 301.0707 [M-Rha-Glc-H_2_O]^+^	233	2	[18]
8	Mv3RG	24.10	C_29_H_35_O_16_^+^	639.1920	639.1921 (0.2)	493.1337 [M-Rha]^+^ 331.0810 [M-Rha-Glc]^+^ 315.0497 [M-Rha-Glc-H_2_O]^+^	235,520	2	[18]
9	Mv3RG5G	10.95	C_35_H_45_O_21_^+^	801.2448	801.2445 (0.3)	639.1932 [M-Glc]^+^ 493.1342 [M-Rha-Glc]^+^ 331.0811 [M-Rha-2Glc]^+^ 315.0500 [M-Rha-Glc-H_2_O]^+^	234	2	[18]
10	Pt3pCouRG5G	20.88	C_43_H_49_O_23_^+^	933.2659	933.2656 (0.3)	771.2119 [M-Glc]^+^ 479.1179 [M-C_9_H_8_O_3_-Rha-Glc]^+^ 317.0655 [M-C_9_H_8_O_3_-Rha-2Glc]^+^	234	2	[18]
IS	Caffeine	8.81	C_8_H_10_N_4_O_2_	195.0877 *	195.0877 (0.0)	138.0662, 109.9588		1	

Note: Entries marked with * indicate [M + H]^+^ ions. Level refers to level of confidence in metabolite identification. The mass error is in ppm.

### 3.2. Relative Changes in Anthocyanin Abundance Across Three Cultivars and Different Organs of TS5

The content and types of anthocyanins are critical in determining plant coloration and function [19]. A detailed analysis of anthocyanin compounds exhibiting significant changes was conducted across four groups (Table S7). Significant features were identified using OPLS-DA, with the threshold for variable importance in projection (VIP) set to 1.0 and the *p*-value set below 0.05. When comparing the two green cultivars (TS2 and TS6), the absolute value of the log_2_ fold change (log_2__FC) ranged from 1.21 to 11.99. The compounds showing an absolute log_2__FC greater than 1 included Mv3A, Cy3G, and peonidin 3-*O*-rutinoside (Pn3RG), whose levels were all reduced in TS6. In a comparison of the green and purple cultivars (TS2 vs. TS5), Cy3G, Pn3G, and Pn3RG were found to have accumulated, with Pn3G showing the most pronounced increase (11.31-fold). In contrast, Mv3A exhibited a reduction. In a comparison of TS5 leaves and flowers (TS5 vs. TS5F), the Mv3A content was found to have increased, whereas the Pn3RG content was reduced. These results are visualized in Figure S2, which shows the upregulated and downregulated metabolites in the flavonoid and anthocyanin metabolic pathways using different color gradients.

### 3.3. Results from the Quantitative Analysis of Anthocyanins

#### 3.3.1. Comparison of Anthocyanin Levels in TS2, TS5, and TS6

Building on the relative abundance comparisons, we performed a quantitative analysis to determine the precise concentrations of key anthocyanins. In the present study, TS5-L exhibited the highest total anthocyanin content among the three cultivars. The predominant anthocyanin was Cy3G, with a concentration of 2470.67 ± 504.49 μg/g, which is consistent with the findings of Ho et al., who previously reported that TS5 contains high levels of anthocyanins, primarily Cy3G and Pn3G [4]. In contrast to previous studies, in this work, we identified seven anthocyanins that had not previously been reported in napier grass, including cyanidin, Mv3A, Pn3RG, Pt3RG, Mv3RG, Mv3RG5G, and Pt3pCouRG5G. Among these, Mv3RG (256.7 ± 68.5 μg/g) and Pn3RG (74.56 ± 24.58 μg/g) were the most abundant in TS5-L (Table 2).

The anthocyanin content in green cultivars (TS2 and TS6) has received relatively little attention in the literature. In this study, among the three cultivars examined, TS2 exhibited a notably high concentration of Mv3A, reaching 703.85 ± 38.01 μg/g, and detectable levels of Cy3G at 31.99 ± 2.80 μg/g and Mv3RG at 43.56 ± 3.12 μg/g. The accumulation of Mv3RG was also evident in TS6, with a concentration of 94.99 ± 15.09 μg/g (Table 2).

#### 3.3.2. Organ-Specific Anthocyanin Distribution in TS5

In the purple cultivar (TS5), the anthocyanin content exhibited a pronounced gradient distribution across different organs, with the highest concentration in the inflorescence, where it was approximately 1.9 times greater than that observed in the leaves. In contrast, the anthocyanin levels dropped sharply in the peel (the epidermis of the stem) and ground stem tissue (Table 2). This distribution pattern is closely related to the physiological functions of specific organs and their strategies for environmental adaptation. The exceptionally high anthocyanin content in the flowers contributes to vivid pigmentation that attracts pollinators and thereby enhances reproductive success [20], while the anthocyanins present in the epidermis and leaves protect plant tissues from damage caused by UV and visible-light exposure [21].

Further analysis revealed that the anthocyanin composition and presence of glycosyl groups also led to organ-specific characteristics in the napier grass samples. In the inflorescence, anthocyanins were predominantly monoglycosylated derivatives such as Cy3G, Mv3A, and Pn3G, accounting for 99.14% of the total anthocyanin content. In contrast, diglycosylated anthocyanins made up only 0.83%. Monoglycosylated anthocyanins are known for their high color intensity [22], which enhances the visual appeal of flowers, thereby attracting pollinators. In comparison, the proportion of diglycosylated anthocyanins, particularly rutinoside derivatives at the 3-OH position, increased significantly in the leaves, reaching 11.30%. Given that rutinoside exhibits greater thermal stability than its monoglycosylated counterparts [23,24], its enrichment in the leaves may enhance their structural stability under prolonged environmental stress.

### 3.4. Compound-Level Insights into Anthocyanin-Related Metabolite Accumulation in Napier Grass

**Figure 5:**
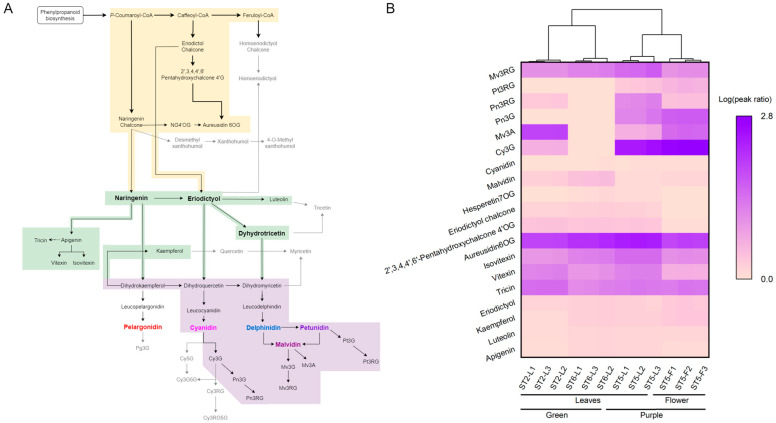
Compound-level visualization of anthocyanin-related metabolites in napier grass. (**A**) Reference pathways of major flavonoids’ and anthocyanins’ biosynthesis used for compound annotation. The pathway diagram was constructed based on the KEGG reference pathways for flavonoid biosynthesis (ko00941) and anthocyanin biosynthesis (ko00942), accessed on 18 April 2025. Only the major metabolic branches relevant to the detected compounds are presented. Background colors indicate different sections: phenylpropanoid and chalcone branch (yellow), core flavonoid pathway (green), and anthocyanin branch (purple). Solid black arrows represent known biochemical conversions; dashed arrows indicate multi-step transformations. Gray arrows denote routes to compounds that were not detected in this study. (**B**) Heatmap showing the relative abundance of flavonoids and anthocyanin-related metabolites mapped in (**A**). This heatmap was generated using OriginPro 2021 with the Clustergram App, applying logarithm transformation and column-wise hierarchical clustering based on the Euclidean distance and average linkage. Each value in the heatmap represents the mean of three technical replicates derived from pooled tissue samples, as described in Section 2.1.

Metabolomics provides a snapshot of the physiological state of an organism at a given time. For the current study, a metabolomics-driven approach was particularly valuable for revealing the structural diversity of anthocyanins in napier grass—a species with limited prior investigation. Napier grass secondary metabolism primarily involves the phenylpropanoid pathway and its downstream branches, including those related to flavonoid and anthocyanin biosynthesis [10]. However, in the context of limited coverage in natural product spectral databases, reference pathways from previous studies play a vital role in supporting metabolite annotation. We adopted a metabolomics approach to comprehensively detect and compare metabolites involved in anthocyanin biosynthesis based on KEGG pathways and known anthocyanin modification patterns observed in plants, as illustrated in Figure 5A. Differences in the accumulation of annotated metabolites were observed across cultivars and organs (Figure 5B).

In this study, we detected metabolites corresponding to multiple known branches of the flavonoid biosynthesis pathway in napier grass. We detected the accumulation of 2′,3,4,4′,6′-pentahydroxychalcone 4′-*O*-β-D-glucoside (pentahydroxychalcone 4′OG) and its downstream product, aureusidin 6-*O*-glucoside (aureusidin 6OG), which are characteristic metabolites of the eriodictyol-type chalcone branch. Aureusidin 6OG was found at relatively high levels in the leaves of all three cultivars, whereas the levels of both pentahydroxychalcone 4′OG and aureusidin 6OG were markedly reduced in the floral tissues. These results indicate that structurally characteristic metabolites of the eriodictyol-type chalcone branch showed higher accumulation in leaves. One additional branch of the flavonoid biosynthetic pathway involves the conversion of feruloyl-CoA into homoeriodictyol. However, the evidence obtained in our study is not sufficient to confirm the presence of intermediates or end-products associated with this route, which are indicated in gray font in Figure 5A.

Another well-characterized intermediate in the flavonoid biosynthesis pathway is naringenin chalcone. According to previous studies and KEGG pathway definitions, naringenin chalcone is formed by the condensation of coumaroyl-CoA and malonyl-CoA, catalyzed by chalcone synthase (CHS), and subsequently converted to naringenin via chalcone isomerase (CHI) [25]. In our study, although several downstream metabolites of naringenin chalcone were detected, naringenin itself was observed in only low levels. Notably, the glycosylated apigenin derivatives, including vitexin and isovitexin, were detected in abundance in napier grass leaves. The relatively high levels of these compounds are consistent with the accumulation of apigenin-derived *C*-glycosides in leaf tissues.

Flavanone 3-hydroxylase (F3H) is an important enzyme involved in anthocyanin biosynthesis, and previous transcriptomic studies have reported significantly higher F3H expression in purple napier grass cultivars compared to that in green ones [10]. However, the profiles of downstream modified anthocyanins revealed considerable structural diversity. Pelargonidin and its glycosylated derivatives were not detected in any of the napier grass samples. In contrast, the major anthocyanin-related compounds detected in the purple cultivar (TS5) and its floral tissues (TS5F) were derivatives of cyanidin, petunidin, malvidin, and peonidin. This composition may account for the reddish-purple to purple pigmentation observed in both leaves and flowers [26], which aligns with the plant’s visible phenotype. The biosynthesis and regulation of these structurally diverse anthocyanin derivatives in napier grass require support from transcriptomic and proteomic investigations. Similarly, in the purple cultivar (TS5), the concentration of Mv3RG was higher than in TS2, whereas the level of Mv3A was markedly lower. Mv3G was not detected in any of the samples. These observations indicate that TS5 primarily accumulated Mv3RG, while TS2 showed relatively higher levels of Mv3A. The absence of detectable Mv3G suggests that it may be subject to alternative forms of modification not captured by our screening compound list or that it may not be formed. These cultivar-specific accumulation patterns may be related to differences in anthocyanin glycosylation and methylation profiles, although the underlying biochemical mechanisms require further investigation.

Anthocyanidins are typically glycosylated at the 3-*O*-position by flavonoid 3-*O*-glucosyltransferase (3GT), which utilizes UDP-glucose as a sugar donor, resulting in the formation of their corresponding 3-*O*-monoglucosides [27]. In our study, high levels of Cy3G were detected in napier grass, whereas other potential downstream derivatives—such as cyanidin 5-*O*-glucoside (Cy5G), cyanidin 3-*O*-glucoside-5-*O*-glucoside (Cy3G5G), and cyanidin 3-*O*-rutinoside (Cy3RG)—were not identified. Accordingly, Cy3G was the major cyanidin-derived compound detected, indicating that glycosylation at the 3-*O*-position may be the predominant modification route observed under our analytical conditions. The absence of detectable Pn3G and Pn3RG suggests that further methylation and rhamnosylation steps, known from other plant systems, were either limited or exhibited traces below the limit of detection in napier grass.

As previously described, monoglycosylated anthocyanins were predominant in the floral tissues of napier grass, with elevated levels of Pn3G and Mv3A and reduced levels of the corresponding rutinoside derivatives, Pn3RG and Mv3RG (Table 2). These tissue-specific differences in glycosylation profiles may reflect variations in anthocyanin modification patterns between leaves and flowers. During flowering, the demand for nitrogen increases, and previous studies have reported that the resorption efficiency of N, P, and K in flowers is positively correlated with the nitrogen content in fresh flowers [28]. According to the photoprotection hypothesis, anthocyanin accumulation may support efficient nitrogen resorption by preventing photo-oxidative stress and circumventing photoinhibition during leaf senescence [29]. These proposed functions indicate a potential link between anthocyanin accumulation, sugar buffering, and nutrient remobilization efficiency in reproductive tissues. Moreover, a previous study reported that inhibition of flavonol biosynthesis increases the incorporation of rhamnose into cell wall polysaccharides, suggesting that a common rhamnose pool is shared between cell wall components and flavonol rhamnosides [30]. In our study, the decline in rutinoside-type anthocyanins observed in floral tissues raises the possibility of competition for rhamnose allocation, although further investigation is needed to confirm this relationship.

## 4. Conclusions

The establishment of an MS^2^ spectral database based on a large number of surrogate samples significantly improved the accuracy of compound identification. Validated through single-laboratory testing, our quantitative analysis also demonstrated good accuracy and precision. Both the quantitative and semi-quantitative results indicated a decreasing trend in the total anthocyanin content of TS5, TS2, and TS6. Notably, the flowers of TS5 exhibited the highest levels of anthocyanins, predominantly represented by Cy3G, Pn3G, and Mv3A. This work represents the first comprehensive profiling of anthocyanin composition across different napier grass organs. The predominance of monoglycosylated anthocyanins in floral tissues highlights their potential as readily accessible sources of bioactive compounds. The application of DIA further broadened metabolite coverage, enabling a pathway-guided, systematic understanding of anthocyanin accumulation patterns in napier grass. These metabolomic data play a crucial role in addressing existing gaps in our knowledge of anthocyanin biosynthesis and structural diversity in this underexplored species. Given its low cost, high biomass yield, and rich diversity of bioactive metabolites, napier grass holds promise as an affordable and scalable source of functional food ingredients.

## Figures and Tables

**Table 2:** Comparison of the anthocyanin content between green and purple napier grass and its distribution across different plant organs.

Compounds	Samples (μg/g)					
	Green		Purple			
	TS2-L	TS6-L	TS5-L	TS5-F	TS5-St	TS5-Sk
Malvidin	Trace	Trace	Trace	Trace	N.D.	Trace
Cyanidin *	Trace	Trace	Trace	Trace	N.D.	Trace
Cy3G	31.99 ± 2.80	N.D.	2470.67 ± 504.49	5019.13 ± 451.39	24.50 ± 1.21	508.39 ± 21.64
Mv3A	703.85 ± 38.01	N.D.	Trace	224.73 ± 20.72	Trace	N.D.
Pn3G	N.D.	N.D.	119.89 ± 26.84	314.97 ± 21.85	1.84 ± 0.09	27.88 ± 1.32
Pn3RG *	Trace	N.D.	74.56 ± 24.58	Trace	Trace	12.10 ± 2.22
Pt3RG *	Trace	N.D.	Trace	Trace	Trace	Trace
Mv3RG *	43.56 ± 3.12	94.99 ± 15.09	256.70 ± 68.50	46.60 ± 6.74	Trace	47.86 ± 6.87
Mv3RG5G *	N.D.	N.D.	N.D.	Trace	N.D.	N.D.
Pt3pCouRG5G *	N.D.	Trace	N.D.	N.D.	N.D.	N.D.

Note: TS2: Taishiu No. 2; TS6: Taishiu No. 6; TS5: Taishiu No. 5; L: L: leaves; F: flowers; St: ground stem tissue; Sk: epidermis of the stem. All values represent the means of three technical replicates subsampled from a pooled composite of approximately 20 individual plants per cultivar and tissue. Entries marked with an * indicate Mv3A equivalent. Trace: concentration of <8 μg/g of sample; N.D.: S/N ≤ 3.

## Data Availability

The raw mass-spectrometry data supporting the findings of this study are not publicly available because of institutional restrictions. However, the processed metabolite dataset is provided in the Supplementary Materials. Additional data may be made available from the corresponding author upon reasonable request and pending institutional approval.

## Supplementary Materials

The following supporting information can be downloaded at: https://www.mdpi.com/article/10.3390/foods14152582/s1, Table S1: Supplementary Information on ZR01; Table S2: Summary of napier grass cultivars used in this study; Table S3: The suspect screening list of anthocyanins; Table S4: Calibration curve of anthocyanins; Table S5: Recovery and repeatability test for anthocyanins; Table S6: The coefficient of variance for intra- and inter- day of contents of anthocyanins; Table S7: Different cumulative metabolites among different comparison groups; Figure S1: The contents control chart of (A) Gy3G; (B) Pn3G; (C) Mv3A and (D) the retention time control chart of Gy3G; (E) Pn3G; (F) Mv3A; Figure S2: A portion of the flavonoid metabolic subnetwork highlighting metabolites with significant cumulative differences among comparison groups.

## Abbreviations

Cy3G	Cyanidin 3-*O*-glucoside
Mv3A	Malvidin 3-*O*-arabinoside
Pn3G	Peonidin 3-*O*-glucoside
Pn3RG	Peonidin 3-*O*-rutinoside
Pt3RG	Petunidin 3-*O*-rutinoside
Mv3RG	Malvidin 3-*O*-rutinoside
Mv3RG5G	Malvidin 3-*O*-rutinoside-5-*O*-glucoside
Pt3pCouRG5G	Petunidin 3-*O*-(*p*-coumaroyl)rutinoside-5-*O*-glucoside
Cy5G	Cyanidin 5-O-glucoside
Cy3G5G	Cyanidin 3-*O*-glucoside-5-*O*-glucoside
Cy3RG	Cyanidin 3-*O*-rutinoside
Pentahydroxychalcone4′OG	2′,3,4,4′,6′-Pentahydroxychalcone 4′-*O*-β-D-glucoside
Aureusidin6OG	Aureusidin 6-*O*-glucoside
Hesperetin7OG	Hesperetin 7-*O*-glucoside
UHPLC-HRMS	Ultra-high-performance liquid chromatography coupled with high-resolution mass spectrometry
NCE	Normalized collision energy
PVDF	Polyvinylidene difluoride
QC	Quality control
OPLS-DA	Orthogonal partial-least-squares discriminant analysis
DIA	Data-independent acquisition
XICs	Extracted ion chromatograms
TS2	Taishiu No. 2
TS5	Taishiu No. 5
TS6	Taishiu No. 6
L	Leaves
F	Flowers
St	Ground tissue of stem
Sk	Epidermis of stem
CHS	Chalcone synthase
CHI	Chalcone isomerase
UDP-Rha	UDP-rhamnose
F3H	Flavanone 3-hydroxylase
