# Supplementary material for: Untargeted Metabolomics Reveals Distinct Anthocyanin Profiles in Napier Grass (Pennisetum purpureum Schumach.) Cultivars
Source: Foods. 2025 Jul 23;14(15):2582. doi: 10.3390/foods14152582 (PMC12345973; doi:10.3390/foods14152582)
Supplement: Supplementary file 1 [file foods-14-02582-s001.zip › Supplementary Tables S1 and S2.pdf]

Supplementary Table S1-S2:

This file contains supplementary tables supporting the main text of the manuscript titled ''Untargeted metabolomics reveals distinct anthocyanin profiles in napier grass (*Pennisetum purpureum* Schumach.) cultivars''.

Table S1. Supplementary Information on ZR01

**Code Name**	ZR01
**IND Number**	160282
**Applicant Company**	Sagittarius Life Science Corp.
**Disclosure Date**	September 11, 2023
**Announcement Title**	*Pennisetum purpureum*-based botanical drug ZR01 approved by the U.S. FDA for Phase I clinical trial in lung cancer
**Source**	https://news.gbimonthly.com/tw/invest/show.php?num=61739

The information presented in this table provides background on ZR01, a *Pennisetum purpureum*-based botanical drug, as disclosed by Sagittarius Life Science Corp.

Table S2. Summary of napier grass cultivars used in this study

<table>
<tr><th>Cultivars of napier grass</th><th>Sampling Location</th><th>Reference URL</th></tr>
<tr><td>TS2 and TS6</td><td>22.5495°N
120.62°E</td><td rowspan="2">https://www.moa.gov.tw/ws.php?id=2503170
https://www.angrin.tlri.gov.tw/grass/Napier56/Napier56.htm</td></tr>
<tr><td>TS5</td><td>25.0154°N,
121.5401°E</td></tr>
</table>

Professor Chwan-Yang Hong provided the samples with verified identification and authenticity.
