# Supplementary material for: Untargeted Metabolomics Reveals Distinct Anthocyanin Profiles in Napier Grass (Pennisetum purpureum Schumach.) Cultivars
Source: Foods. 2025 Jul 23;14(15):2582. doi: 10.3390/foods14152582 (PMC12345973; doi:10.3390/foods14152582)
Supplement: Supplementary file 1 [file foods-14-02582-s001.zip › Supplementary Tables S4-S7 and Figures S1 and S2.pdf]

Supplementary Information

This file contains supplementary figures, tables, and detailed methods supporting the main text of the manuscript titled ‘’Untargeted metabolomics reveals distinct anthocyanin profiles in napier grass (*Pennisetum purpureum* Schumach.) cultivars’’.

Contents:

- Table S4. Calibration curve of anthocyanins
- Table S5. Recovery and repeatability test for anthocyanins
- Table S6. The coefficient of variance for intra- and inter- day of contents of anthocyanins
- Table S7. Different cumulative metabolites among different comparison groups
- Figure S1. The contents control chart of (A) Gy3G; (B) Pn3G; (C) Mv3A and (D)the retention time control chart of Gy3G; (E) Pn3G; (F) Mv3A
- Figure S2. A portion of the flavonoid metabolic subnetwork highlighting metabolites with significant cumulative differences among comparison groups

Table S4. Calibration curve of anthocyanins

Compounds	Linear range (μg/g)	Calibration curve	R^2
Mv3A	8.0 - 3200.0	y = 0.2292x + 4.1327	0.9992
Cy3G	1.6 - 400.0	y = 0.2216x - 0.3344	0.9986
Pn3G	1.6 - 400.0	y = 0.2614x - 0.3198	0.9972

Mv3A: Malvidin 3-*O*-arabinoside; Cy3G: Cyanidin 3-*O*-glucoside; Pn3G: Peonidin 3-*O*-glucoside.

Table S5. Recovery and repeatability test for anthocyanins

Compounds	Spike level (μg/g)	Recovery[a] (%)	Spike level (μg/g)	Recovery[a] (%)	Spike level (μg/g)	Recovery[a] (%)
Mv3A	4.0	85.5±7.4	400.0	105.8±1.1	1600.0	100.3±1.5
Cy3G	4.0	105.5±5.5	40.0	104.0±4.5	400.0	113.7±2.7
Pn3G	4.0	102.1±1.8	40.0	100.6±2.5	400.0	108.1±3.8

[a]Recovery expressed as mean ± standard deviation, n = 3.

Mv3A: Malvidin 3-*O*-arabinoside; Cy3G: Cyanidin 3-*O*-glucoside; Pn3G: Peonidin 3-*O*-glucoside.

Table S6. The coefficient of variance for intra- and inter- day of contents of anthocyanins

Compounds	Contents, mean (mg/g)	Coefficient of Variation (%)					
		Intra-day					Inter-day
		day 1	day 2	day 3	day 4	day 5	
Mv3A	0.236	0.005	0.004	0.002	0.003	0.007	2.778
Cy3G	4.827	0.100	0.072	0.030	0.138	0.091	2.654
Pn3G	0.301	0.006	0.007	0.003	0.004	0.004	2.397

Mv3A: Malvidin 3-*O*-arabinoside; Cy3G: Cyanidin 3-*O*-glucoside; Pn3G: Peonidin 3-*O*-glucoside.

Table S7. Different cumulative metabolites among different comparison groups

Group	Compounds	Class	VIP	p_value	log2_FC	Type
**TS2 vs TS6**	Mv3A	Monoglycoside-anthocyanins	2.64	1.078E-08	-11.99	Down
	Cy3G	Monoglycoside-anthocyanins	1.50	4.277E-06	-10.36	Down
	Tricin	flavonoids	1.07	1.070E-03	-0.36	Down
	Pn3RG	Diglycoside-anthocyanins	1.06	3.625E-06	-9.36	Down
**TS2 vs TS5**	Cy3G	Monoglycoside-anthocyanins	2.07	4.080E-06	1.96	Up
	Mv3A	Monoglycoside-anthocyanins	1.75	5.677E-06	-1.58	Down
	Pn3G	Monoglycoside-anthocyanins	1.69	1.915E-05	11.31	Up
	Pn3RG	Diglycoside-anthocyanins	1.39	8.429E-05	1.86	Up
	Isovitexin	flavonoids	1.12	1.883E-06	0.65	Up
	Mv3RG	Diglycoside-anthocyanins	1.11	6.601E-04	0.61	Up
**TS5 vs TS5F**	Mv3A	Monoglycoside-anthocyanins	1.60	3.238E-07	1.21	Up
	Pn3RG	Diglycoside-anthocyanins	1.45	3.926E-05	-1.37	Down
	Vitexin	flavonoids	1.32	1.833E-04	-0.92	Down
	Mv3RG	Diglycoside-anthocyanins	1.24	8.640E-07	-0.59	Down
	Isovitexin	flavonoids	1.12	8.680E-04	-0.48	Down
	Pn3G	Monoglycoside-anthocyanins	1.11	4.175E-05	0.43	Up

Acquisition and integration of total ion chromatograms were performed using Xcalibur 4.0 software (Thermo Fisher Scientific). Extracted ion chromatograms (XICs) corresponding to the target metabolites were obtained and integrated. Peak area ratios ($Area_{anal}$ / $Area_{IS}$) were normalized by logarithm transformation prior to multivariate analysis. The processed data were subsequently imported into the OmicShare

online platform (http://www.omicshare.com/tools) for orthogonal partial least squares discriminant analysis (OPLS-DA). Metabolites with VIP ≥ 1 and $p < 0.05$ were considered statistically significant. log_2_FC: log_2 fold change. Mv3A: Malvidin 3-*O*-arabinoside; Cy3G: Cyanidin 3-*O*-glucoside; Pn3G: Peonidin 3-*O*-glucoside; Pn3RG: Peonidin 3-*O*-rutinoside; Mv3RG: Malvidin 3-*O*-rutinoside.

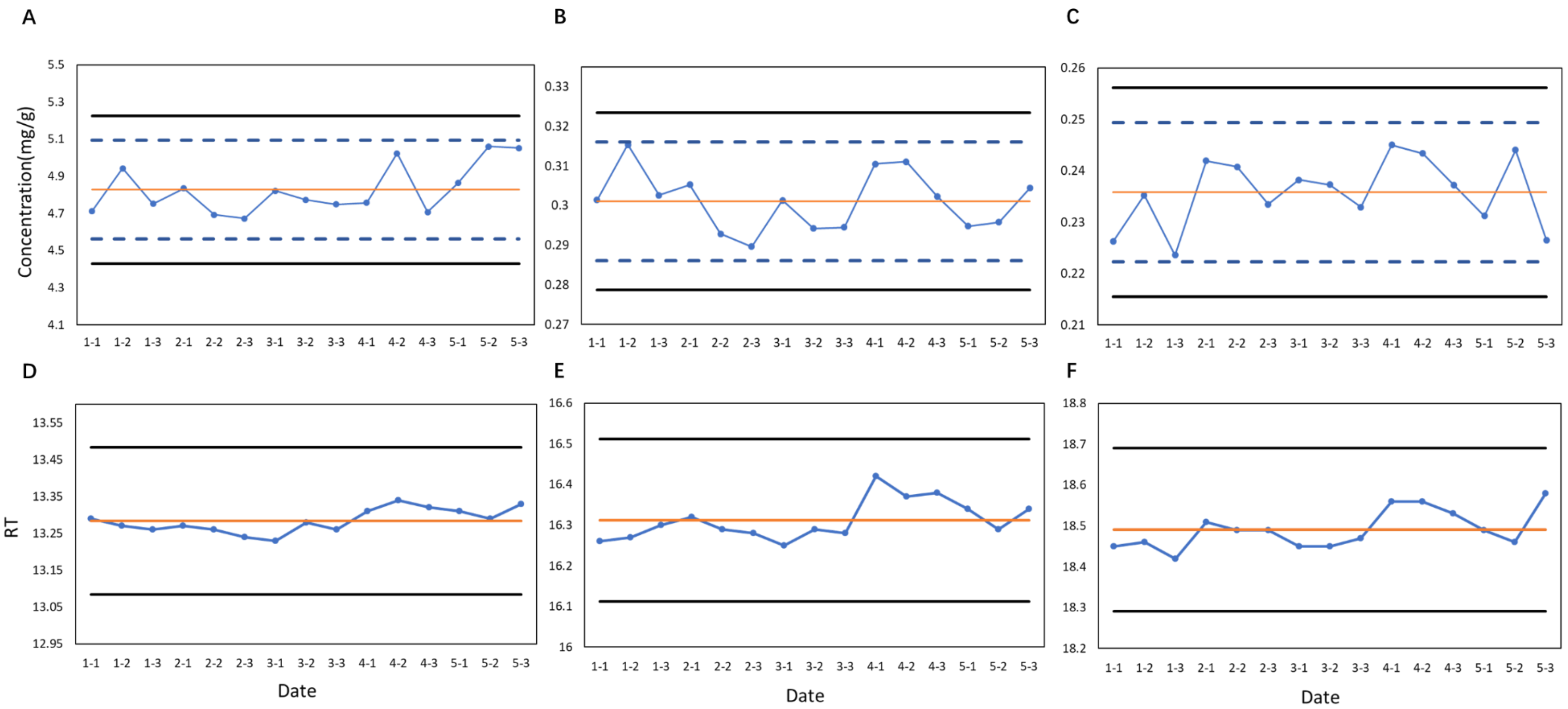

Figure S1. The contents control chart of (A) Gy3G; (B) Pn3G; (C) Mv3A and (D)the retention time control chart of Gy3G; (E) Pn3G; (F) Mv3A. Mv3A: Malvidin 3-*O*-arabinoside; Cy3G: Cyanidin 3-*O*-glucoside; Pn3G: Peonidin 3-*O*-glucoside.

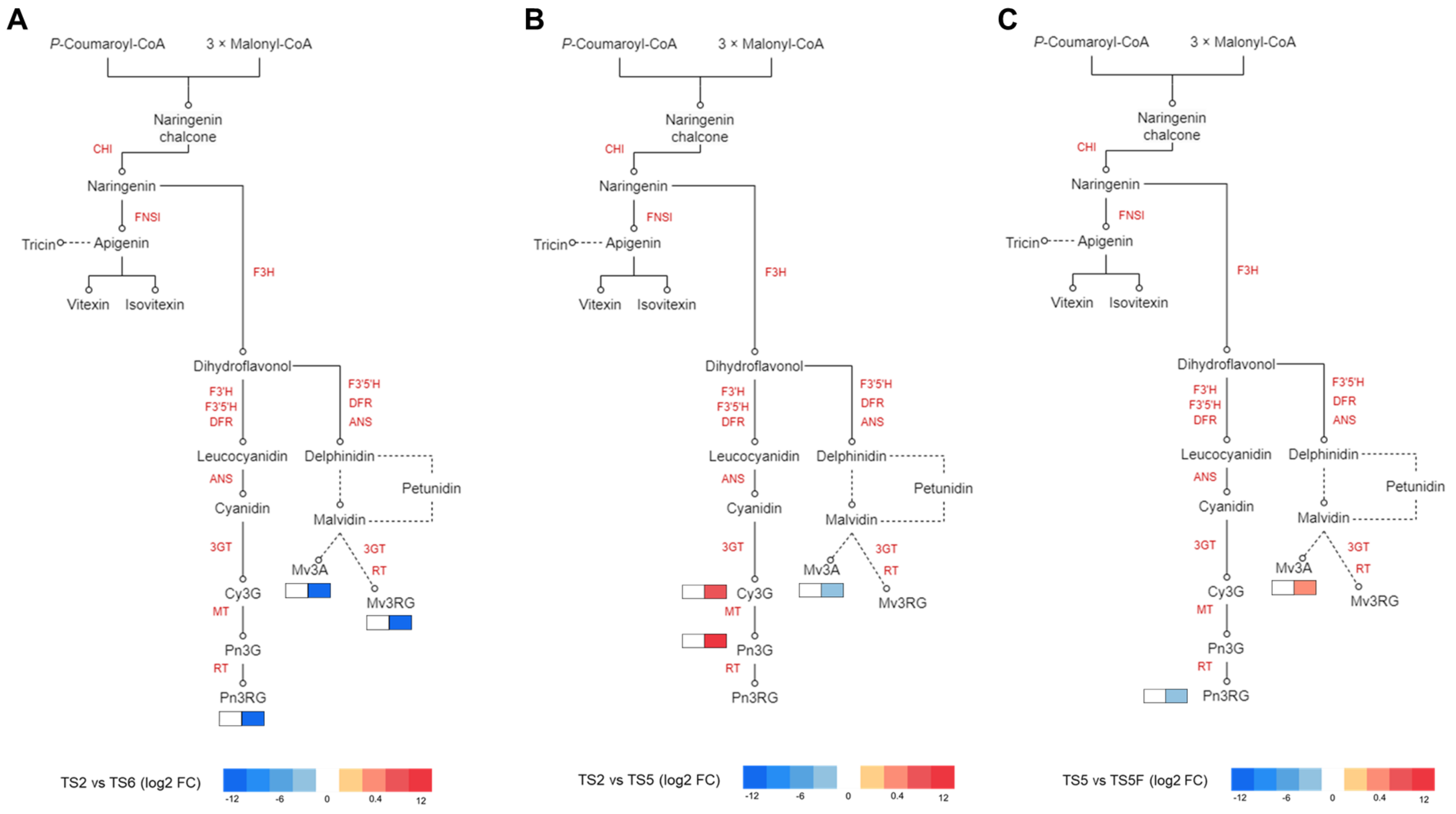

Figure S2. A portion of the flavonoid metabolic subnetwork developed based on KEGG pathways, highlighting metabolites with significant cumulative differences among comparison groups. Differential metabolites between the two sample groups. Color gradients indicate relative content differences (log_2 FC). Acquisition and integration of total ion chromatograms were performed using Xcalibur 4.0 software (Thermo

Fisher Scientific). Extracted ion chromatograms (XICs) corresponding to the target metabolites were obtained and integrated. Peak area ratios (Areaanal / AreaIS) were normalized by logarithm transformation prior to multivariate analysis. The processed data were subsequently imported into the OmicShare online platform (http://www.omicshare.com/tools) for orthogonal partial least squares discriminant analysis (OPLS-DA). Metabolites with VIP $\geqslant$ 1 and $p < 0.05$ were considered statistically significant. Mv3A: Malvidin 3-*O*-arabinoside; Cy3G: Cyanidin 3-*O*-glucoside; Pn3G: Peonidin 3-*O*-glucoside; Pn3RG: Peonidin 3-*O*-rutinoside; Mv3RG: Malvidin 3-*O*-rutinoside.
